# Haplotype Construction Using Embryos as Probands of the Pathogenic Variations in EXT1, CUL3, and HBA

**DOI:** 10.1155/humu/2768102

**Published:** 2026-03-31

**Authors:** Defeng Shu, Yi Liu, Xiaoli Wang, Tao Liang, Zubo Wu

**Affiliations:** ^1^ Department of Obstetrics and Gynecology, Union Hospital, Tongji Medical College, Huazhong University of Science and Technology, Wuhan, China, hust.edu.cn; ^2^ Department of Clinical Laboratory, Union Hospital, Tongji Medical College, Huazhong University of Science and Technology, Wuhan, China, hust.edu.cn; ^3^ Department of Pediatrics, Union Hospital, Tongji Medical College, Huazhong University of Science and Technology, Wuhan, China, hust.edu.cn

**Keywords:** CUL3, EXT1, haplotype, HBA, preimplantation genetic testing

## Abstract

Preimplantation genetic testing (PGT) represents a crucial strategy in the prevention of monogenic disorders, ensuring that only embryos free from these genetic conditions are implanted during assisted reproductive technologies. By analyzing the type of haplotypes of the variation of the probands or the carriers, we can significantly enhance the diagnostic precision of PGT. We presented a clinical strategy that uses embryos as probands to construct haplotypes; this innovative approach has successfully delineated the haplotypes associated with pathogenic variations in key genes, such as HBA (encoding hemoglobin subunit alpha), EXT1 (involved in exostoses), and CUL3 (a gene related to various developmental disorders). Ten embryos in three families were tested, all are diagnosed whether with the deletion variations or not by haplotype construction, Sanger sequencing, or PCR. Importantly, we compared SNP results with haplotype analysis by pedigree linkage or long reading sequence. This method can be considered when family members are incomplete and haplotype construction is otherwise unfeasible other than long reading sequencing.

## 1. Introduction

Preimplantation genetic testing (PGT) is a crucial technique for preventing the transmission of monogenic diseases, serving as an alternative to prenatal diagnosis. Given the embryonic stage′s characteristic of having a limited number of cells with low DNA concentration, direct detection is difficult. Typically, amplifying the entire genome is necessary to fulfill the objectives of the test. However, this process of whole‐genome amplification (WGA) is prone to allele dropout (ADO), a phenomenon that, if occurring at the pathogenic locus, could lead to diagnostic errors and missed diagnoses in PGT [1]. To mitigate the risk of such missed diagnoses due to ADO, reassessment for the presence of pathogenic variations in the embryo through haplotyping becomes essential. For the establishment of haplotypes, the most frequently utilized markers are either short tandem repeat (STR) sites or single‐nucleotide polymorphism (SNP) sites [2]. Generally, the haplotype harboring the pathogenic variation is often deduced by testing the proband along with the parents, providing a familial genetic context.

However, in many cases, family history may not provide sufficient information, or the proband may carry a de novo pathogenic mutation, complicating the establishment of haplotypes. Long‐read sequencing technology offers a solution by directly sequencing long DNA fragments from the proband to establish haplotypes, but this approach is expensive [3]. An alternative strategy involves using embryos or gametes as probands, along with genetic testing of both parents, to determine haplotypes [4–6]. This method faces challenges such as amplification dropout at the variant site, which can render the test unsuccessful and hinder haplotype construction. Currently, there is limited research on the widespread applicability of this method in PGT for monogenic diseases (PGT‐M) across various genetic disorders.

The EXT1 gene is linked with multiple osteochondromas. Previous studies have shown PGT‐M using haplotypes established through family members prevented the inheritance of pathogenic variants and results in the birth of phenotypically healthy fetuses [7]. The *CUL3* gene, associated with neurodevelopmental disorders and pseudohypoaldosteronism, is inherited in an autosomal dominant manner, with its pathogenic variants leading to neurodevelopmental disorders [8–10]. However, PGT‐M targeting variants in the *CUL3* gene have not been reported. *α*‐Thalassemia, a prevalent monogenic disease, has been broadly investigated for its prevention through PGT‐M [11]. Although there have been instances of employing embryos as probands for haplotype construction, the specific types of variants and details of haplotype construction remain underreported [6]. This study highlights the successful construction of haplotypes using embryos as probands involving pathogenic variants in three different genes (*EXT1*, *CUL3*, and *HBA*) within the scope of assisted reproductive technology and the subsequent PGT of embryos. Method uses haplotype information of embryos to improve the accuracy of diagnosis and compared SNP results with haplotype analysis by pedigree linkage or long‐reading sequence.

## 2. Materials and Methods

### 2.1. Patients

**Figure 1:**
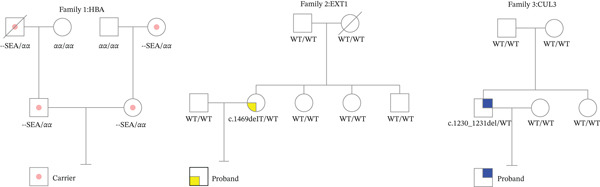
Pedigrees of the three mutations of the three families: the proband, carrier, and the genotype of the members are shown in the figure.

The research data were collected from patients at the Reproductive Center of Tongji Medical College, Huazhong University of Science and Technology Affiliated Union Hospital, in 2023. In Family 1, a couple undergoing preconception carrier screening were both found to be carriers of the *α*‐thalassemia SEA heterozygote. The father of the male partner was deceased, and his mother did not carry this mutation, precluding the possibility of constructing his haplotype through family genetic history. In Family 2, the female partner was diagnosed with hereditary multiple osteochondromas. Genetic testing revealed a de novo pathogenic heterozygous variant c.1469delT (p. L490Rfs∗9) in the *EXT1* gene, with neither of the female partner′s parents carrying this variant, nor any family history of the disease, thus making family‐based haplotype construction unfeasible. In Family 3, the male partner, serving as the proband, exhibited mild intellectual disability. Whole‐exome sequencing uncovered a heterozygous likely pathogenic variant c.1230_1231del (p. Leu412fs) in the *CUL3* gene, which was absent in both parents of the male partner, indicating another case of a de novo mutation. The information of the three families is listed in Table 1 and Figure 1.

**Table 1:** : The clinical information of the three families.

Case ID	Female age	Female karyotype	Male age	Male karyotype	Gene	Female mutation sites	Male mutation sites	Evaluation by ACMG	Family history	Cycle	Embryos tested
1	33	46,XX	33	46,XY	*HBA*	‐‐SEA	‐‐SEA	P	None	1	4
2	32	46,XX	32	46,XY	*EXT1*	c.1469delT(p.Leu490Rfs∗9)	Wild type	P	None	1	4
3	33	46,XX	23	46,XY	*CUL3*	Wild type	c.1230_1231del(p.Leu412fs)	LP	None	1	2

Furthermore, for the two PGT‐M families where haplotypes were successfully constructed through pedigree linkage and long‐read sequencing, we performed embryos as probands in these two cases. The haplotypes derived from embryos as probands were compared with those constructed by pedigree linkage and long‐read sequencing to verify their accuracy and differences. The family information used for validation is presented in Table 2.

**Table 2:** The genetic information of the two families which constructed the haplotype by pedigree linkage sequencing and long‐reading sequence.

	Gene	Variation	Gene‐related disease	Male genotype	Female genotype	Aborted fetus genotype	Number of the embryo tested
Pedigree linkage analysis	GALC	c.1901 T > C	Krabbe disease	Heterozygote	Heterozygote	Homozygote	20
	ABCC8	c.1887del	Diabetes mellitus	Wild type	Heterozygote	Wild type	
Long‐reading sequence	NFIX	c.303dupC	Malan syndrome	Wild type	Heterozygote	Null	9

The *α*‐thalassemia Southeast Asian (SEA) deletion is a type of mutation on the *α*‐globin gene cluster which is prevalent in SEA. The mutation deletes four genes and one pseudogene: *μ*‐globin gene (*HBM*), pseudo *α*‐globin gene (*HBAP1*), *α*‐globin 2 gene (*HBA2*), *α*‐globin 1 gene (*HBA1*), and *θ*‐globin gene (*HBQ1*). The length of the SEA deletion type is 19,304 bp. The deletion was mapped onto assembly genome sequence GRCh38.p7 (GenBank assembly accession: GCA_000001405.22); it showed the position of the deletion from 165 397 to 184 700 [12].

### 2.2. Biopsy Collection

Patients′ oocytes were fertilized through intracytoplasmic sperm injection following oocyte retrieval. Subsequently, embryos were cultured in vitro until reaching the blastocyst stage, typically on Day 5 or Day 6 postfertilization. Biopsies were performed on each blastocyst, extracting 5–10 trophectoderm (TE) cells per specimen. These biopsied TE cells were then transferred into EP tubes containing cell lysis buffer, followed by the next detection.

### 2.3. WGA and Mutation Detection

TE biopsies were performed, and WGA was carried out using a universal sample processing kit, ChromSwiftTM (XK‐028, Yikon Genomics) for gene sequencing according to the manufacturer′s instructions for gene sequencing. WGA products were fragmented for library construction and sequenced on the Illumina NextSeq 550 platform to analyze the ploidy of each embryo. Three gigabytes of data or 9 million reads were required for each WGA product to perform copy number variation (CNV) analysis and SNP haplotyping. Sanger sequencing of mutation loci was also conducted to determine the genotype of the embryos.

### 2.4. CNV Detection

Raw sequencing reads underwent quality control and adapter trimming using fastp (v0.26.0). The processed reads were aligned to the human reference genome (GRCh37/hg19) with BWA‐MEM2 (v2.2.1), after which PCR duplicates were marked and removed using Sambamba (v0.8.0).

For copy number analysis, the genome was segmented into 400‐kb sliding windows with a 200‐kb step size, generating approximately 30,000–35,000 bins. Regions with poor mappability, including satellite repeats, centromeres, telomeres, and other high‐complexity sequences, were excluded. Read counts per window were normalized for GC content bias using LOESS regression. The normalized read depths were then compared against a reference panel of control samples to calculate log_2_ copy number ratios and *Z*‐scores, with sex‐specific adjustments applied where appropriate.

Putative CNVs were identified by segmenting the log_2_ ratio profiles using the circular binary segmentation (CBS) algorithm within the DNAcopy R package. Segments with log_2_ ratios exceeding ± 0.25 were reported as CNVs. The reporting criteria included whole‐chromosome aneuploidies, whole‐arm events, and segmental CNVs no smaller than 4 Mb (supported by at least 20 consecutive bins). For the detection of intermediate copy number states (often referred to as mosaicism in other contexts), segments were required to be at least 10 Mb in length with an estimated level exceeding 30%.

### 2.5. Haplotype Analyses Using the SNPs Based on Microarray

Entering the ovulation induction cycle, a successful biopsy and WGA were achieved, alongside utilizing WGA products from embryos and DNA extracted from both parents′ peripheral blood for SNP detection. For SNP site analysis, data from high‐throughput sequencing were mapped to the human reference genome (hg19). The detection process included embryo WGA, purification of amplification products, extraction of DNA from family samples, DNA amplification, DNA fragmentation, precipitation of DNA fragments, DNA resuspension, hybridization, chip washing, extension, staining, and scanning with the Infinium Asian Screening Array (Cat# 20016317, Illumina, San Diego, California).

Analysis was performed using the ChomGO software (v1.8; Yikon). Only heterozygous SNPs within 5‐Mb upstream and downstream of pathogenic mutations will be used for phasing, avoiding the influence of ADO and recombination. For parental haplotype phasing, genetic information of pedigrees, such as sibling embryos or other family members, is needed. A likelihood‐based haplotyping approach using a hidden Markov model (HMM) and the dynamic programming Viterbi algorithm was used to determine the most likely haplotype configuration of parents. The transition probability of haplotype state changes between consecutive loci in the HMM was calculated from the recombination fraction obtained from the genetic distance map of the 1000 Genomes Project Phase 3 references (https://www.internationalgenome.org/). Additionally, direct assessment of mutation sites in the embryo WGA products was conducted using sequencing technologies, such as Sanger sequencing. This analysis helped determine whether the mutation was inherited from the father or the mother by comparing the mutation sites of the embryo with those of the parents to ascertain the source of the SNP haplotype of the embryo′s mutation. If the embryo inherits the mutation from the father, the matching SNP haplotype in the embryo indicates the disease‐causing gene mutation haplotype, whereas the other haplotype is considered normal. Similarly, if the mutation is inherited from the mother, the SNP haplotype in the embryo that matches the mother′s is identified as the carrier of the disease‐causing gene mutation haplotype, with the alternate haplotype being normal. Here is an example in Table 3.

**Table 3:** The example to construct the haplotype using embryo as proband.

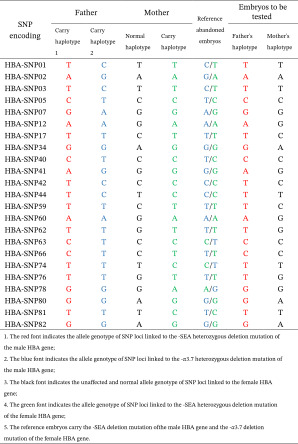

#### 2.5.1. Phasing and Haplotype Linkage Analysis using Nanopore Sequencing

Raw electrical signals from the MinION platform were base‐called into FASTQ format reads using Guppy (v5.0.16). To ensure data quality, reads were filtered with NanoFilt (v2.8.0) to remove low‐quality sequences (Phred score ≤ 7) and short reads (< 1000 bp), and 50 bp were trimmed from each read terminus. Filtered reads were aligned to the human reference genomes (GRCh37, GRCh38, and T2T‐CHM13) using Minimap2 (v2.26) with the parameters: ‐ax map‐ont ‐L ‐‐MD ‐Y ‐t 20. Resulting SAM files were converted to sorted BAM files using SAMtools (v1.2) for downstream processing. Structural variant (SV) calling was performed on the aligned BAM files using Sniffles (‐t 12 ‐‐min_support 1 ‐‐num_reads_report ‐1). Initial SV calls were filtered based on high‐quality supporting reads and corroborative karyotype reports.

For variant calling and haplotype phasing, the PEPPER‐WhatsHap‐DeepVariant pipeline (r0.7‐gpu) was employed with the ‐‐ont_r9_guppy5_sup ‐g ‐‐phased_output ‐t 12 parameters. This generated a phased VCF file containing single‐nucleotide variants and indels. A custom Python (v3.10.1) script was then used to extract and classify haplotype information within the target genomic regions. Finally, a likelihood‐based haplotyping approach, implemented via a HMM, was applied to determine the most probable haplotype configuration for embryos and other non‐proband samples.

## 3. Results

### 3.1. Genetic Variant Detection in Parents and Embryos

**Figure 2:**
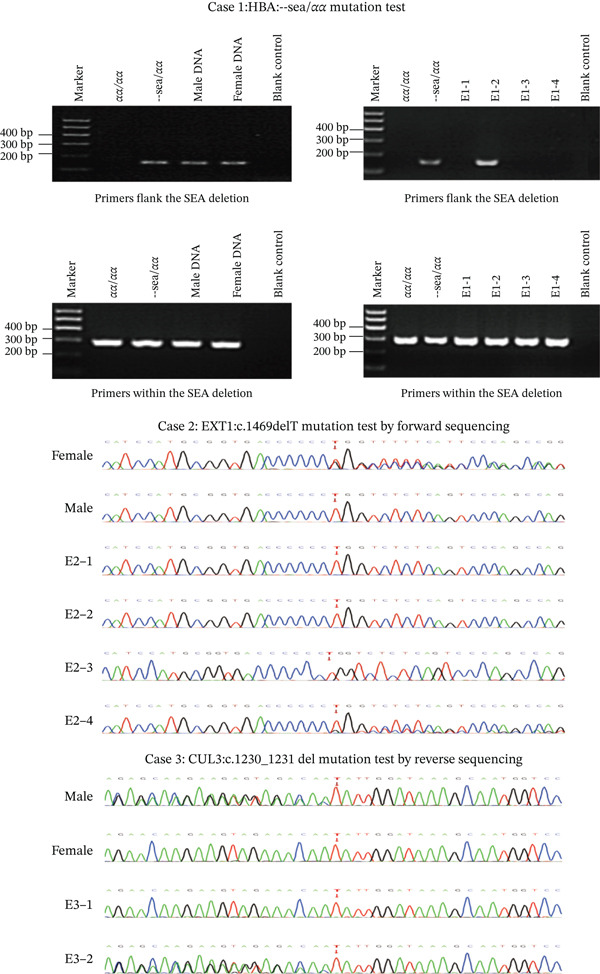
The Gap‐PCR results of ‐‐SEA mutations in the parents and the embryos in Family 1. Male DNA and female DNA are the carriers from the marker, *αα*/*αα*, ‐‐sea/*αα*, and blank control. There was no DNA amplification because the deletion is too wide with the primers flank the SEA deletion. The Sanger sequencing showed the mutations tests in Families 2 and 3 and their embryos.

Utilizing Sanger sequencing and Gap‐PCR methodology, we successfully identified genetic variants in both parental samples and embryos. Through an analysis of targeted genes variations associated with hereditary disorders, we discerned specific variants contributing to the genetic landscape of each individual and embryos in Figure 2.

### 3.2. Haplotype Establishment Through Embryo

**Figure 3:**
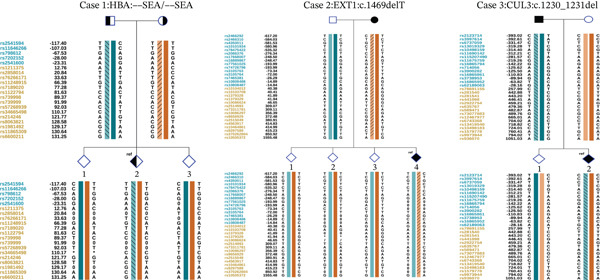
Haplotype of HBA, EXT1, CUL3 using the embryos as probands. There are only three embryos in Case 1 haplotype because the CNV test of the E1‐4 is triploid by SNP analysis; it is difficult to construct the haplotype in triploid.

We established haplotypes for the investigated genetic loci by proband as embryo with the variation. This enabled the elucidation of parental allele inheritance patterns within the embryonic genome, facilitating the precise selection of embryos decreasing the risks of ADO in Figure 3.

### 3.3. Detection of CNVs in Embryonic Chromosomes

**Figure 4:**
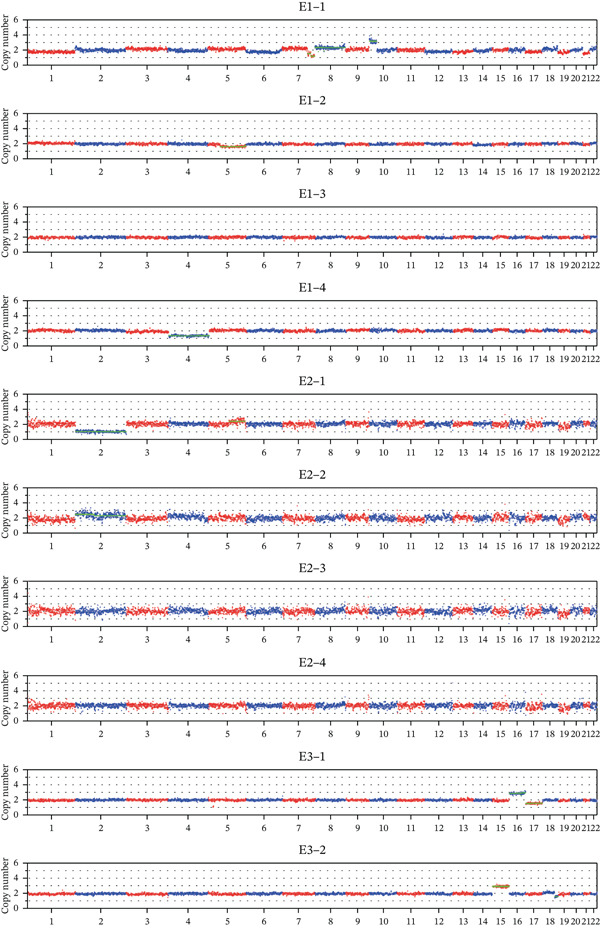
The CNV results of the embryos in these three families.

All embryos underwent CNV analysis to detect chromosomal abnormalities. This revealed significant proportions of embryos with abnormalities, emphasizing the necessity of CNV screening alongside mutation detection in Figure 4. All the detecting and clinical results are showed in Table 4.

**Table 4:** The clinical results of every embryo: +: CNV abnormal; −: CNV normal; EAP: embryo as proband. WT: wild type.

Summary of the PGT‐M results of the embryos									
Case ID	Mutation gene	Mutation site	Cycle	Embryo ID	CNV	Mutation detection	Proband	Haplotype analysis	Clinical outcomes
1	*HBA*	‐‐SEA/‐‐SEA	1	E1‐1	+	WT/WT	EAP(E1‐2)	WT/WT	Abandoned
			1	E1‐2	+	Carrier	EAP(E1‐2)	Carrier	Abandoned
			1	E1‐3	−	WT/WT	EAP(E1‐2)	WT/WT	Ectopic pregnancy
			1	E1‐4	+	WT/WT	EAP(E1‐2)	Failure	Abandoned

2	*EXT1*	c.1469delT(p.Leu490Rfs∗9)	1	E2‐1	+	WT	EAP(E2‐4)	WT	Abandoned
			1	E2‐2	+	WT	EAP(E2‐4)	WT	Abandoned
			1	E2‐3	−	WT	EAP(E2‐4)	WT	Implantation failure
			1	E2‐4	−	Carrier	EAP(E2‐4)	Carrier	Abandoned

3	*CUL3*	c.1230_123	1	E3‐1	+	WT	EAP(E3‐2)	WT	Abandoned
		1del(p.Leu412fs)							
			1	E3‐2	+	Carrier	EAP(E3‐2)	Carrier	Abandoned

### 3.4. Amniocentesis Results of One Case

**Figure 5:**
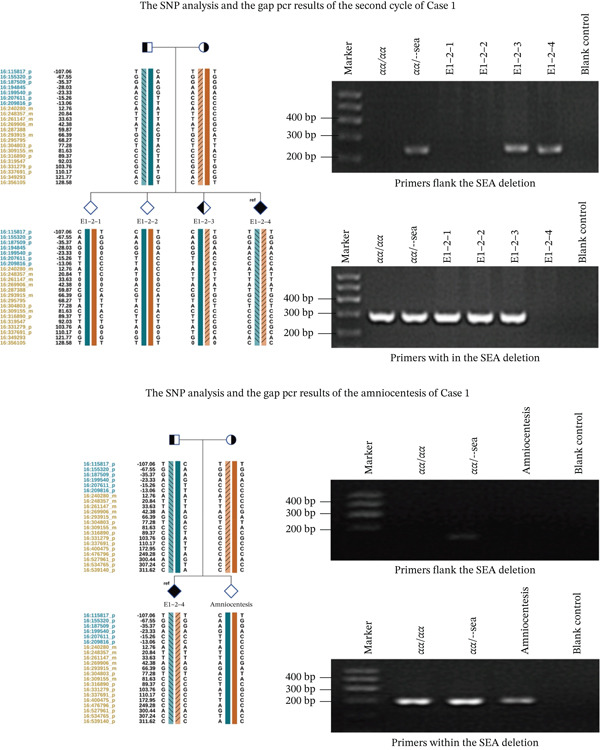
The SNP analysis and the Gap‐PCR results of the embryos and the amniocentesis of Case 1.

Case 1 underwent the second cycle of PGT‐M, one embryo is the CNV normal and without the HBA mutation, the embryo was transferred and delivered a healthy baby, the embryo results and the amniocentesis results are shown in Figure 5.

### 3.5. SNP Results of Different Methods

**Figure 6:**
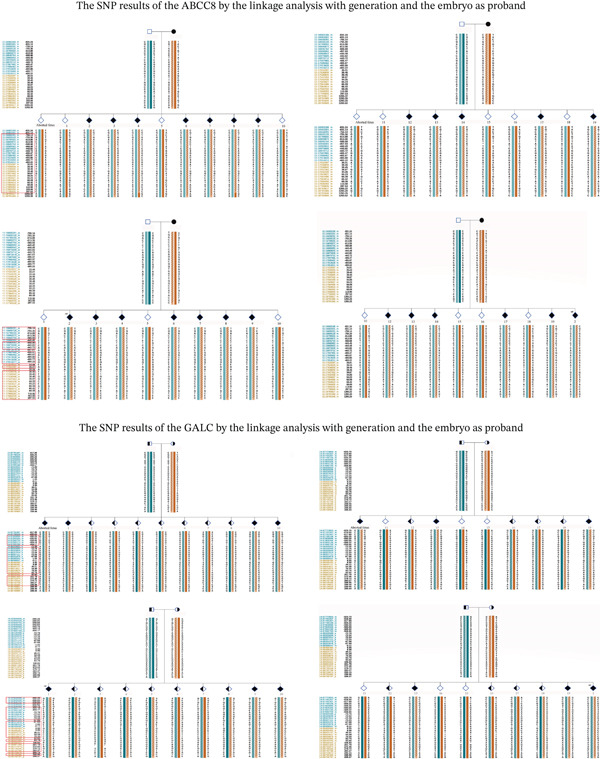
The block labeled “aborted fetus” shows haplotypes constructed through family linkage analysis, with no PCR amplification performed in the testing process. The block labeled “ref” displays haplotypes constructed using the embryo as the proband, where PCR amplification was performed during testing. The SNP loci highlighted in red boxes represent the same sites captured by both methods.

**Figure 7:**
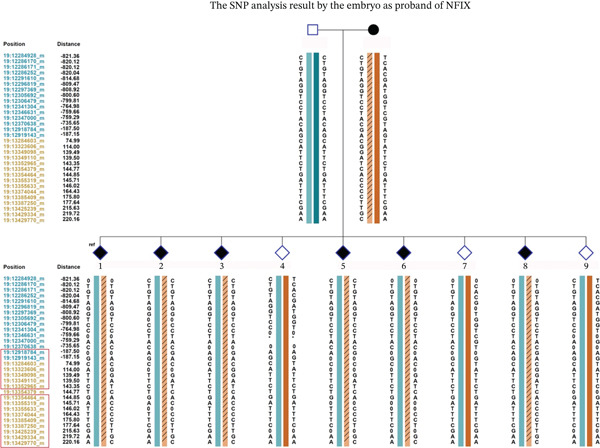
The block labeled “ref” displays haplotypes constructed using the embryo as the proband. The SNP loci highlighted in red boxes represent the same sites captured by long‐reading sequence and NGS.

We performed a comparative analysis between the SNP loci used for haplotype construction through embryo proband–family linkage and those obtained via long‐read sequencing, with the results presented in Figures 6 and 7. The haplotye constructed by long ‐read sequencing is presented in Table 5.

**Table 5:** The closest SNP information captured by long‐reading sequence to the mutation site of NFIX: The red SNP sites are the haplotype of the pathogenic variation of NFIX.

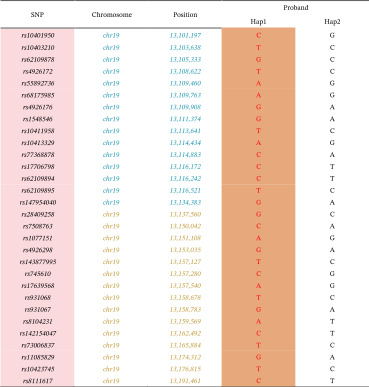

## 4. Discussion

Our study demonstrates successful embryo‐based haplotype construction for pathogenic variants in three different genes, providing relevant experience and reference for future haplotype construction for similar variants. This method effectively shortens the cycle for patients to undergo PGT‐M, expands the applicability of PGT‐M, and reduces the financial burden. At the meantime, we found that the embryo as proband method can provide more SNP locus than the pedigree linkage analysis, although long‐reading sequence can capture more SNP locus, but not all the loci can be tested on embryos with NGS, these findings are not reported in previous research (Table 5).

The establishment of haplotypes is crucial for PGT‐M, effectively avoiding misdiagnosis caused by ADO [13]. The occurrence of ADO is random, and no amplification method can completely avoid the probability of ADO [14]. Currently, SNP and STR sites are commonly used for linkage analysis to determine haplotypes, typically between two generations of family members. However, it becomes challenging to establish haplotypes through family members or de novo mutations. Successful haplotype construction can be achieved using long‐reading sequencing, enabling PGT‐M [15, 16]. Methods of haplotype construction using embryos as probands have been reported for PKD1 and *α*‐thalassemia, resulting in the birth of healthy infants. We report three cases of successful embryo‐based haplotype construction for different gene variants, providing more evidence for the feasibility of this method for broader clinical applications. However, there are still risks associated with using embryos for haplotype construction, including ADO due to sequence‐rich GC regions and variations in amplification systems [17]. Additionally, detecting specific mutations during the embryonic stage, especially with the shortcomings of second‐generation sequencing technology, such as dynamic mutations and complex rearrangements, remains challenging [18, 19]. Specialized detection methods for specific mutations during the embryonic stage, such as Gap‐PCR targeting the deletion mutation in *α*‐thalassemia, have been demonstrated in our study. Furthermore, insufficient embryo numbers or the presence of triploids or haploidy in the region of the pathogenic variant can lead to failed haplotype construction.

Haplotype construction typically involves identifying relevant SNP sites within two megabases upstream and downstream of the pathogenic variant. However, even with this approach, there is a possibility of gene recombination within the two‐megabase region, potentially leading to diagnostic errors. Although this possibility is low, patients should still be informed of the associated risks. Subsequent prenatal diagnosis can mitigate adverse outcomes resulting from these risks. In addition to searching for SNP sites around the variant, some researchers have suggested that haplotypes can also be successfully constructed based on family member information if the variant is in the identity‐by‐state (IBS) region [20]. Even so, if gene recombination occurs in the mutation region [21], it can lead to the failure of diagnosing whether the embryo carries the mutation through haplotype analysis. Therefore, combining haplotype analysis with direct cell detection can improve the diagnostic rate of embryos.

Currently, haplotype construction is widely applied with family‐based linkage analysis, long‐read sequencing technologies, and Phbol‐seq techniques [22–24]. Additionally, the use of cell‐free DNA for diagnosis of genetic disease has also been reported [25, 26], The accuracy of performing PGT‐M using culture medium is lower than that of traditional cell biopsy methods. Embryo as proband technology eliminates the need for additional sample testing beyond parental samples. Furthermore, our research demonstrates that even when haplotypes have been successfully constructed using other methods, incorporating embryo as proband data analysis can provide additional SNP loci in the target region. These newly added SNP loci may assist in determining recombination sites, though further clinical data are needed to support this hypothesis.

However, our study′s limitation is the lack of many successful pregnancy outcomes and follow‐up information due to CNV abnormalities. This further illustrates the necessity of detecting CNVs when performing single‐gene disorder detection [27]. Additionally, the sample size of the clinical data is too small, and more data is needed to support the feasibility of this method, as well as the potential risks and countermeasures. Moreover, although these variants are rated as pathogenic or likely pathogenic according to ACMG guidelines [28], necessary functional experiments to verify the impact of the variants on protein expression and their manifestation in the organism are required.

Nevertheless, detecting and constructing haplotypes for special types of variants during the embryonic stage requires the development of new technologies and further research. Exploring effective ways to reduce ADO and improve PCR efficiency remains an ongoing endeavor.

## 5. Conclusion

Using embryos as probands in preimplantation genetic testing is a feasible and cost‐effective approach. However, the presence of trisomy or monosomy in the pathogenic variant regions of the embryo can lead to diagnostic challenges if the mutation is not detected in the embryo, particularly when ADO is encountered in the testing process.

## Author Contributions

Yi Liu supervised the genetic consultation, diagnostic evaluation, and clinical management of the cases. Xiaoli Wang and Tao Liang collaborated on writing the manuscript. Yi Liu contributed to clinical support. Defeng Shu and Zubo Wu conducted the literature review, wrote the manuscript, and coordinated its submission. All authors contributed to the final version of the article. Tao Liang and Zubo Wu contributed equally to this work.

## Funding

This study was supported by the Natural Science Foundation Program of Hubei Province (2022CFB181, 2024AFB672), the Free Innovation Pre‐Research Fund of Union Hospital of Tongji Medical College, Huazhong University of Science and Technology (2021XHYN059, 2023XHYN052), and the National Key R&D Program of China (2023YFC2705505).

## Disclosure

All authors approved the final version of the article.

## Ethics Statement

This study was conducted in accordance with the principles outlined in the Declaration of Helsinki. All patients involved in this study were fully informed about the associated risks and provided their informed consent to participate. This study was approved by the institutional Ethics Committee of Union Hospital of Tongji Medical College, Huazhong University of Science and Technology.

## Conflicts of Interest

The authors declare no conflicts of interest.

## Data Availability

The datasets used in this article are not publicly accessible to protect the anonymity of participants and patients. Researchers interested in accessing the datasets may contact the corresponding author for further information and consideration. This policy is implemented to ensure the confidentiality and privacy of the individuals included in the study.
